# Neural selectivity for communicative auditory signals in Phelan-McDermid syndrome

**DOI:** 10.1186/s11689-016-9138-9

**Published:** 2016-02-23

**Authors:** A. Ting Wang, Teresa Lim, Jesslyn Jamison, Lauren Bush, Latha V. Soorya, Teresa Tavassoli, Paige M. Siper, Joseph D. Buxbaum, Alexander Kolevzon

**Affiliations:** Seaver Autism Center for Research and Treatment, Icahn School of Medicine at Mount Sinai, One Gustave L. Levy Place, Box 1230, New York, NY 10029 USA; Department of Psychiatry, Icahn School of Medicine at Mount Sinai, New York, NY USA; Department of Neuroscience, Icahn School of Medicine at Mount Sinai, New York, NY USA; Friedman Brain Institute, Icahn School of Medicine at Mount Sinai, New York, NY USA; Department of Psychiatry, Rouge Valley Health System, Toronto, Canada; Department of Communication Sciences and Disorders, Northwestern University, Evanston, IL USA; Rush University Medical Center, Chicago, IL USA; Department of Genetics and Genomic Sciences, Icahn School of Medicine at Mount Sinai, New York, NY USA; Mindich Child Health and Development Institute, Icahn School of Medicine at Mount Sinai, New York, NY USA; Department of Pediatrics, Icahn School of Medicine at Mount Sinai, New York, NY USA

## Abstract

**Background:**

Phelan-McDermid syndrome (PMS), a neurodevelopmental disorder caused by deletion or mutation in the *SHANK3* gene, is one of the more common single-locus causes of autism spectrum disorder (ASD). PMS is characterized by global developmental delay, hypotonia, delayed or absent speech, increased risk of seizures, and minor dysmorphic features. Impairments in language and communication are one of the most consistent characteristics of PMS. Although there is considerable overlap in the social communicative deficits associated with PMS and ASD, there is a dearth of data on underlying abnormalities at the level of neural systems in PMS. No controlled neuroimaging studies of PMS have been reported to date. The goal of this study was to examine the neural circuitry supporting the perception of auditory communicative signals in children with PMS as compared to idiopathic ASD (iASD).

**Methods:**

Eleven children with PMS and nine comparison children with iASD were scanned using functional magnetic resonance imaging (fMRI) under light sedation. The fMRI paradigm was a previously validated passive auditory task, which presented communicative (e.g., speech, sounds of agreement, disgust) and non-communicative vocalizations (e.g., sneezing, coughing, yawning).

**Results:**

Previous research has shown that the superior temporal gyrus (STG) responds selectively to communicative vocal signals in typically developing children and adults. Here, selective activity for communicative relative to non-communicative vocalizations was detected in the right STG in the PMS group, but not in the iASD group. The PMS group also showed preferential activity for communicative vocalizations in a range of other brain regions associated with social cognition, such as the medial prefrontal cortex (MPFC), insula, and inferior frontal gyrus. Interestingly, better orienting toward social sounds was positively correlated with selective activity in the STG and other “social brain” regions, including the MPFC, in the PMS group. Finally, selective MPFC activity for communicative sounds was associated with receptive language level in the PMS group and expressive language in the iASD group.

**Conclusions:**

Despite shared behavioral features, children with PMS differed from children with iASD in their neural response to communicative vocal sounds and showed relative strengths in this area. Furthermore, the relationship between clinical characteristics and neural selectivity also differed between the two groups, suggesting that shared ASD features may partially reflect different neurofunctional abnormalities due to differing etiologies.

**Electronic supplementary material:**

The online version of this article (doi:10.1186/s11689-016-9138-9) contains supplementary material, which is available to authorized users.

## Background

Genetic liability to autism spectrum disorder (ASD) is now understood to be due in part to rare genetic variants. Deletions and mutations of the *SHANK3* gene, located at 22q13.3, are rare but are still one of the more common single-locus causes of ASD. The loss of one functional copy of *SHANK3* results in Phelan-McDermid syndrome (PMS), characterized by global developmental delay, moderate to severe intellectual disability (ID), delayed or absent speech, hypotonia, and ASD or ASD features [1]. Studies estimate the prevalence of *SHANK3* deficiency in ASD and ID to be as high as ~0.5 to 2 %, on the same order as fragile X syndrome, tuberous sclerosis, and Rett syndrome [2]. Soorya et al. [3] recently evaluated 32 individuals with PMS using gold-standard diagnostic instruments for autism and found that 84 % of the sample met the criteria for ASD, suggesting that PMS is one of the more highly penetrant genetic causes of ASD.

The *SHANK3* gene codes for a scaffolding protein that aids in the assembly of postsynaptic signaling components at glutamatergic synapses [4]. *Shank3* rodent models have begun to examine how changes at the molecular and cellular levels are associated with structural brain abnormalities as well as specific behaviors. *Shank3-*deficient mice show deficits in synaptic function and plasticity, with impaired maintenance of long-term potentiation [5–9]. Abnormalities in dendritic spine morphology, including decreased spine density, have been observed in the hippocampus [8] and striatum [10]. At the behavioral level, *Shank3*-deficient mice often exhibit motor deficits, reduced social interactions, and altered ultrasonic vocalizations [5, 6, 8, 9]. Interestingly, recent data demonstrate that insulin-like growth factor (IGF-1) reverses the electrophysiological and motor deficits seen in the *Shank3* heterozygous mice at doses identical to those used clinically [6]. Furthermore, in vitro assays have shown that synaptic deficits in neurons from patients with PMS can be corrected by restoring *SHANK3* expression or treatment with IGF-1 [11]. However, despite this promising preclinical evidence that synaptic alterations in *SHANK3* deficiency may represent therapeutic targets, to date, there is a dearth of data, particularly in humans, on the abnormal neural systems underlying PMS. 

Magnetic resonance imaging (MRI) scans performed as part of clinical profiling suggest that common findings may include thinning of the corpus callosum, white matter abnormalities, ventricular dilation, cerebellar vermis hypoplasia, and arachnoid cysts [12–17]. No studies to date reporting structural abnormalities have included comparison data. One study examined cerebral blood flow and observed hypoperfusion of the left temporal pole and amygdala in 8 children with PMS relative to 13 children with idiopathic ID [16], but the comparison group was not characterized (i.e., no information was provided on mean age, cognitive abilities, etc.), making the findings difficult to interpret. No studies have examined brain function using functional MRI (fMRI) in PMS to the best of our knowledge.

One of the most consistent behavioral features of PMS is impaired language development, including delayed or absent speech. Soorya et al. [3] reported that none of the 32 participants evaluated used phrase speech on a daily basis and only 19 % used single words to communicate consistently. Individuals with PMS also show deficits in using and understanding gestures and other forms of nonverbal communication, such as eye contact and facial expression—impairments also associated with idiopathic ASD (iASD). Critical to the development of communication skills is the ability to detect communicative signals in the environment. Previous neuroimaging studies have established that the cortex along the superior temporal sulcus and gyrus (STS/STG) plays an essential role in processing speech and other vocal sounds [18–21]. Shultz and colleagues [19] found that a region of the STS/STG responds selectively to communicative vocalizations (e.g., speech, laughter, sounds of agreement/disagreement) over and above vocalizations that are non-communicative (e.g., throat clearing, coughing, yawning), lending support to the notion that the STS/STG is sensitive not just to voices but also to the communicative significance that the sounds convey [22]. Typically developing children attend selectively to salient communicative cues such as voices and speech sounds from infancy [23]; children with ASD, however, do not show the same early preference [24, 25]. Perhaps not surprisingly, individuals with ASD do not appear to show selective activity in the STS/STG for vocal sounds [26]. Little is known about social attention in PMS or the neural architecture that supports it.

In this study, we used fMRI to examine brain activity in response to communicative and non-communicative sounds in children with PMS and those with iASD. We sought to examine whether children with PMS and iASD have neural circuitry specialized to detect the communicative significance of vocalizations. Another goal of the study was to examine brain-behavior relationships in PMS and iASD. We investigated the extent to which clinical symptoms, such as autism symptom severity, attention to social stimuli, and language level, were related to selective brain activity for communicative sounds in each group. By examining the similarities and differences in brain function and brain-behavior relationships between children with ASD with and without *SHANK3* deficiency, we aim to better understand the clinical heterogeneity observed in ASD.

## Methods

### Participants

Eleven children and adolescents with PMS (six boys, five girls; mean age = 7.9 ± 3.9 years) and nine children with iASD (all boys, mean age = 9.5 ± 3.9 years) participated in the study. PMS participants were recruited through the Phelan-McDermid Syndrome Foundation and ongoing studies at the Seaver Autism Center for Research and Treatment at the Icahn School of Medicine at Mount Sinai. *SHANK3* deletions or mutations were confirmed for PMS participants using chromosomal microarray (CMA) or Sanger sequencing. For individuals with iASD, pathogenic deletions and mutations were ruled out using CMA and whole exome sequencing. Parents provided written informed consent according to the guidelines of the Icahn School of Medicine Institutional Review Board, which approved all study procedures.

Participants received a comprehensive clinical evaluation including medical and psychiatric evaluation, ASD diagnostic assessments (Autism Diagnostic Observation Schedule, second edition (ADOS-2 [27]) and Autism Diagnostic Interview–revised (ADI-R [28])), a developmental assessment (Mullen Scales of Early Learning (MSEL [29])), and a measure of adaptive functioning (Vineland Adaptive Behavior Scales, second edition [30]). A measure of social orienting [31] was also conducted on a subgroup of eight participants with PMS as part of a clinical trial at the Seaver Autism Center. These eight participants were scanned and assessed during baseline characterization for the trial. Social orienting impairment is the failure to orient spontaneously to naturally occurring social stimuli in the environment and may reflect a core deficit of ASD. For the social orienting task, developed by Dawson and colleagues [31], the participant was seated across from an examiner and engaged in play with a toy or book. A second examiner walked around the room delivering social (i.e., patting legs, calling the child’s name, humming, snapping fingers) or non-social sounds (i.e., blowing a whistle, a timer, phone ring, car horn). Orienting was defined as turning the head or eyes toward the sound within 15 s.

All participants in both groups were nonverbal or minimally verbal, with fewer than 20 words used during the ADOS, module 1. All iASD participants met ADOS, ADI-R, and DSM-5 criteria for ASD. All PMS participants met ADOS criteria and 10 of 11 PMS participants (91 %) met consensus clinical judgment for ASD based on all available information (i.e., ADOS, ADI-R, and DSM-5). Developmental quotient (DQ) scores were calculated by dividing the developmental age equivalent averaged across receptive language, expressive language, visual reception, and fine motor subscales on the MSEL by the child’s chronological age (CA) and multiplying by 100, as is commonly done to avoid floor and ceiling effects [32, 33]. Similarly, language and nonverbal quotients were calculated by averaging the receptive and expressive age equivalents for language and fine motor and visual reception age equivalents for the nonverbal domain, dividing by CA and multiplying by 100. Participant characteristics are presented in Table 1.

**Table 1:** Participant characteristics

	PMS	iASD	*p* value
	Mean (SD)	Mean (SD)	
Age, years	7.9 (3.9)	9.5 (3.9)	0.37
DQ	21 (14)	35 (25)	0.13
Language quotient	17 (14)	31 (23)	0.13
Nonverbal quotient	24 (15)	39 (28)	0.15
Mullen receptive language AE (months)	14 (10)	30 (9)	0.001
Mullen expressive language AE (months)	11 (9)	25 (12)	0.005
ADOS total	18 (6)	20 (4)	0.43
ADOS social affect	16 (5)	15 (SD)	0.72
ADOS repetitive behavior	3 (2)	5 ± 2	0.007
ADI communication	12 (3)	13 ± 5	0.27
ADI social interaction	20 (7)	24 ± 5	0.16
ADI repetitive behavior	4 (2)	7 ± 2	0.02
Vineland adaptive behavior composite	52 (11)	55 ± 10	0.59

*SD* standard deviation, *DQ *developmental quotient, *Mullen* Mullen Scales of Early Learning, *AE* age equivalents, *ADOS* Autism Diagnostic Observation Schedule, second edition, *ADI* Autism Diagnostic Interview, revised

### fMRI paradigm

Participants were presented with auditory stimuli consisting of communicative and non-communicative sounds [19]. Categories of communicative sounds were adult-directed speech, infant-directed speech, and communicative vocalizations (e.g., sounds of agreement, disagreement, disgust); categories of non-communicative sounds were sounds of walking, water, and non-communicative vocalizations (e.g., sneezing, coughing, hiccups). Each sound category was presented five times for a total of 30 activation blocks separated by 12 s of rest. Blocks were presented in a pseudorandom order so that no sound category occurred more than twice in a row. Detailed information on the sound properties and acoustic features of the stimuli can be found in Shultz et al. [19].

### Image acquisition

To minimize movement, all participants were scanned under propofol sedation administered and monitored by a pediatric anesthesiologist. Images were acquired on a Siemens 3 T Allegra scanner at the Icahn School of Medicine at Mount Sinai. Functional images were collected using a standard echo-planar sequence (repetition time = 2.0 s, echo time = 25 ms, field of view = 220 mm, flip angle = 60°, matrix size = 64 × 64, voxel size = 3.4 × 3.4 × 4 mm, 34 slices). High-resolution, T1-weighted anatomical images used for registration were acquired with a 3D MPRAGE sequence (repetition time = 2.5 s, echo time = 4.38 ms, field of view = 210 mm, flip angle = 8°, matrix size = 256 × 256, voxel size = 0.82 × 0.82 × 0.82 mm, 208 slices).

### Image analysis

Data were preprocessed and analyzed using SPM5 (Wellcome Department of Cognitive Neurology, London; http://www.fil.ion.ucl.ac.uk/spm/). For each participant, functional images were (1) realigned to correct for head motion using a least squares approach and a six-parameter rigid body transformation, (2) coregistered to his/her high-resolution anatomical sequence, (3) spatially normalized into the MNI-152 standard template (Montreal Neurological Institute) using a 12-parameter affine transformation, and (4) smoothed with an 8-mm full-width half-maximum isotropic Gaussian kernel.

For all participants, within each group, condition effects were estimated according to the general linear model using a box-car reference function with a 6-s delay to compensate for the hemodynamic response lag, high-pass filtering (128 s), and a first-degree autoregressive model (AR(1)) to estimate intrinsic autocorrelations in the data. The resulting contrast images were entered into second-level analyses using a random effects model. For each group, one-sample *t* tests were implemented for each contrast of interest (e.g., communicative vs. non-communicative sounds). Between-group differences were examined using two-sample *t* tests. Whole-brain multiple regression analyses were used to examine neural activity associated with ASD symptom severity, as measured by ADOS social affect and repetitive behavior scores, as well as receptive and expressive language ability. For the PMS group, a simple regression analysis was used to identify regions of activity associated with social orienting.

We used a combined voxel intensity and cluster extent threshold to correct for multiple comparisons and guard against type I error. Assuming an individual voxel level threshold of *p* < 0.05, a Monte Carlo simulation was conducted to determine the appropriate voxel contiguity threshold. The simulation takes into account the image resolution parameters and the 8-mm FWHM smoothing to estimate cluster-level false positive rates (see Slotnick and Schacter [34] for a description of procedures). After 1000 iterations, a cluster extent of 194 contiguous resampled voxels (2 × 2 × 2 mm^3^) was indicated as necessary to correct for multiple voxel comparisons across the whole brain at *p* < 0.05.

## Results

The PMS and iASD groups did not differ significantly in chronological age, DQ, ADOS total, or Vineland Adaptive Behavior Composite (see Table 1). However, the PMS group had significantly lower receptive and expressive age equivalents on the MSEL than the iASD, although overall language quotient was not significantly different. In addition, the iASD group showed significantly higher (more impaired) ADOS scores in the restricted and repetitive behaviors domain than the PMS group, although the social affect domain and total ADOS scores did not differ significantly between groups. On the social orienting task, children with PMS turned their head or eyes toward a social sound an average of 59 % of the time (standard deviation (SD) = 33 %) and toward a non-social sound for 65 % of the trials (SD = 33 %).

**Fig. 1:**
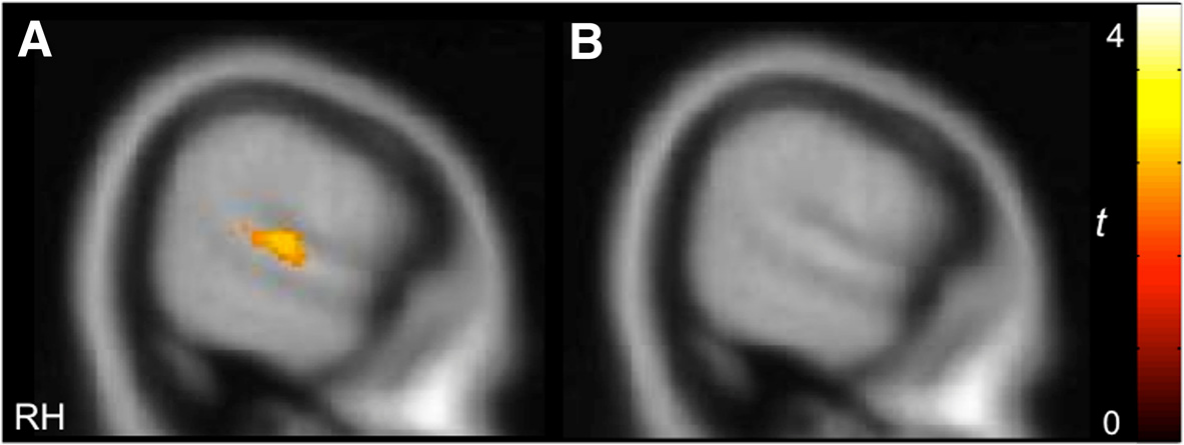
Differential activity in the STG elicited by communicative vs. non-communicative vocalizations in the PMS group (**a**), but not the iASD group (**b**) (*p* < 0.05, *k* > 194, corrected)

As expected, both groups showed activity in the primary auditory cortex in response to all sounds relative to rest. In the PMS group, listening to communicative vocalizations yielded selective activity compared to non-communicative vocalizations in the right STG in a region comparable to that previously observed in typically developing adults [19] (MNI coordinates: *x =* 64, *y =* -36, *z =* 12; *Z =* 1.89, cluster size (*k*) = 238 voxels, *p* < 0.05, corrected; Fig. 1a). In contrast, the iASD group did not show differential activity in the STG for communicative relative to non-communicative sounds (Fig. 1b). Between-group comparisons confirmed that selective STG activity was indeed significantly greater in the PMS relative to iASD group (*x =* 64, *y =* −38, *z =* 12; *Z =* 2.64, *k =* 6021). Furthermore, greater differential activity for the PMS group was also observed in other regions involved in social cognition and language, including the medial prefrontal cortex (MPFC: *x =* −18, *y =* 50, *z =* 10; *Z =* 3.44, *k =* 6021), posterior cingulate cortex (PCC: *x =* −16, *y =* −46, *z =* 16; *Z =* 3.16, *k =* 5081), and inferior frontal gyrus (IFG: *x =* −40, *y =* 22, *z =* −6; *Z =* 3.42, *k =* 598) (Additional file 1: Figure S1). The iASD group showed greater selective activity for communicative sounds in more posterior visual and subcortical regions, including the inferior and middle occipital gyri, dorsal striatum, and cerebellum. Peak coordinates of significant activity across the whole brain are presented in Table 2.

**Table 2:** Peaks of activation for communicative vs. non-communicative vocalizations

			PMS group				iASD group				PMS > iASD				iASD > PMS			
Anatomical region	BA	H	*x*	*y*	*z*	*t*	*x*	*y*	*z*	*t*	*x*	*y*	*z*	*t*	*x*	*y*	*z*	*t*
Superior temporal gyrus	22	R	64	–36	12	2.09					64	–38	12	2.96				
	38	L	–46	24	–20	3.22					–44	22	–20	2.54				
	42	R	64	–20	10	3.00												
Middle temporal gyrus	39	R													38	–50	16	2.67
	39	R																
Superior frontal gyrus	6	L	–6	6	62	2.65					–8	–18	66	2.81				
Medial prefrontal cortex	9	R									18	44	28	3.94				
	10	L	–16	50	4	2.02					–18	50	10	4.16				
	10										0	56	–6	2.71				
Anterior cingulate cortex	24	L	–8	–6	42	3.22	–8	28	12	4.56					–4	24	14	3.05
	24	R													12	8	36	2.93
	32	L	–12	40	12	2.72	–14	36	14	3.77	–18	48	0	4.11				
	32	R													22	26	34	2.80
Inferior frontal gyrus	47	L	–38	26	–12	3.38					–40	22	–6	4.12				
	45	L	–38	24	16	3.90												
	45	R	36	30	14	4.13												
	44	L									–42	0	24	2.78				
	44	R	34	24	20	2.81					50	18	18	4.12				
Middle frontal gyrus	46	R									44	36	10	2.92				
	9	R									50	34	20	2.89				
Insula		L	–34	26	0	4.55					–44	–8	16	3.68				
		R	38	18	2	2.18	28	28	0	6.01								
Posterior cingulate cortex	23	L									–16	–46	16	3.72				
	31	R									14	–46	48	3.42				
Cuneus	17	L					–4	–86	10	6.09								
	18	L									–18	–88	10	3.27				
	18	R					4	–78	18	2.93								
Lingual gyrus	18	L	–22	–72	–8	3.94												
	19	L	–22	–66	8	3.08												
Middle occipital gyrus	18	L					–20	–84	22	5.20								
	19	R													42	–82	8	2.98
Inferior occipital gyrus	18	R					26	–82	–10	3.05								
Cerebellum		L					–40	–48	–36	5.46	–22	–76	–44	3.36				
		R					32	–68	–44	5.51								
Caudate nucleus		L					–8	–2	20	5.36					–2	2	18	4.56
		R					12	–4	20	5.92					20	24	12	2.95
Putamen		R	28	16	2	2.77												

**Fig. 2:**
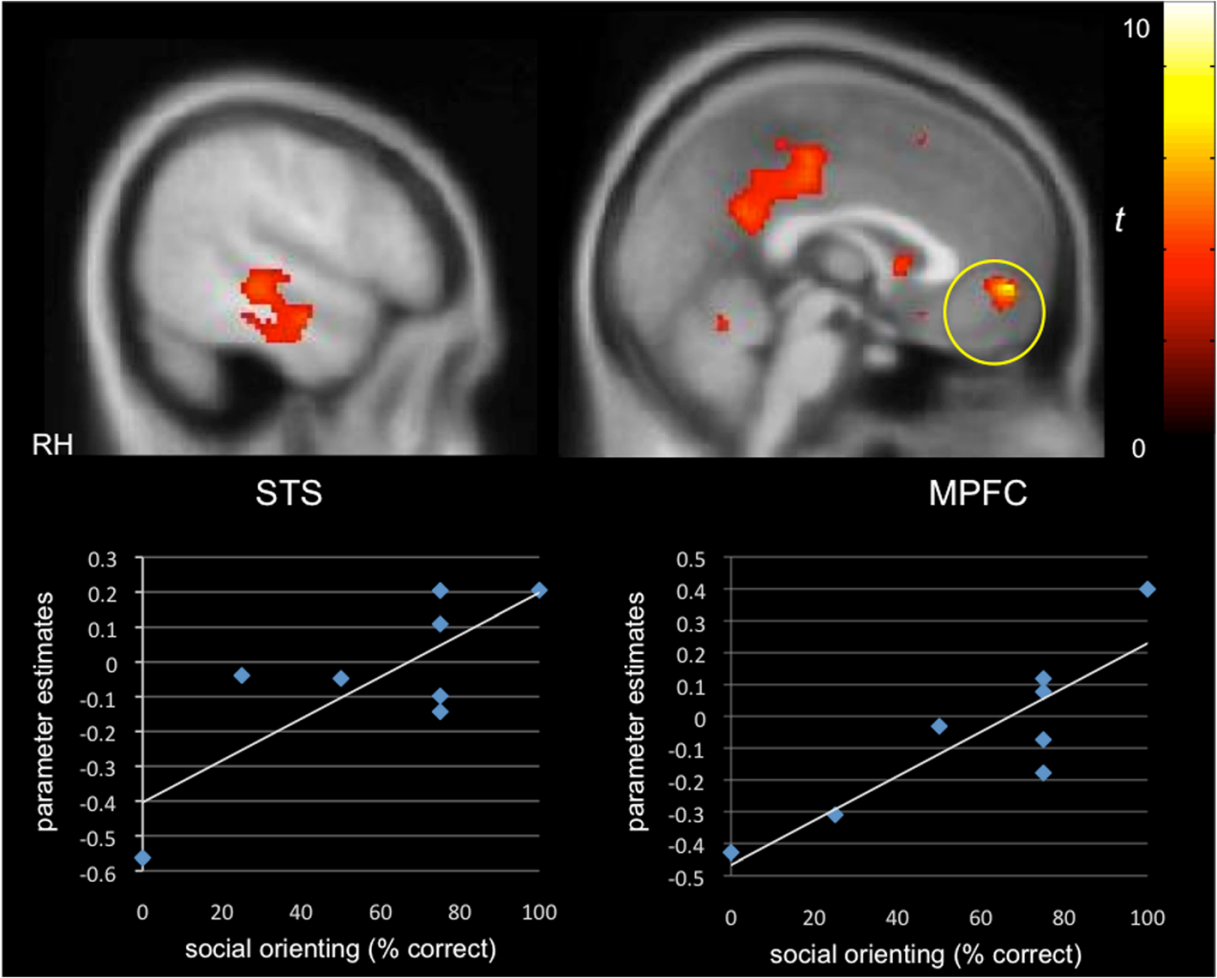
Selective activity for communicative vs. non-communicative sounds as a function of social orienting abilities in PMS. A positive correlation was observed between differential “social brain” activity (e.g., cortex along the STS, MPFC) and social orienting skills in the PMS group (*p* < 0.05, *k* > 194, corrected)

**Fig. 3:**
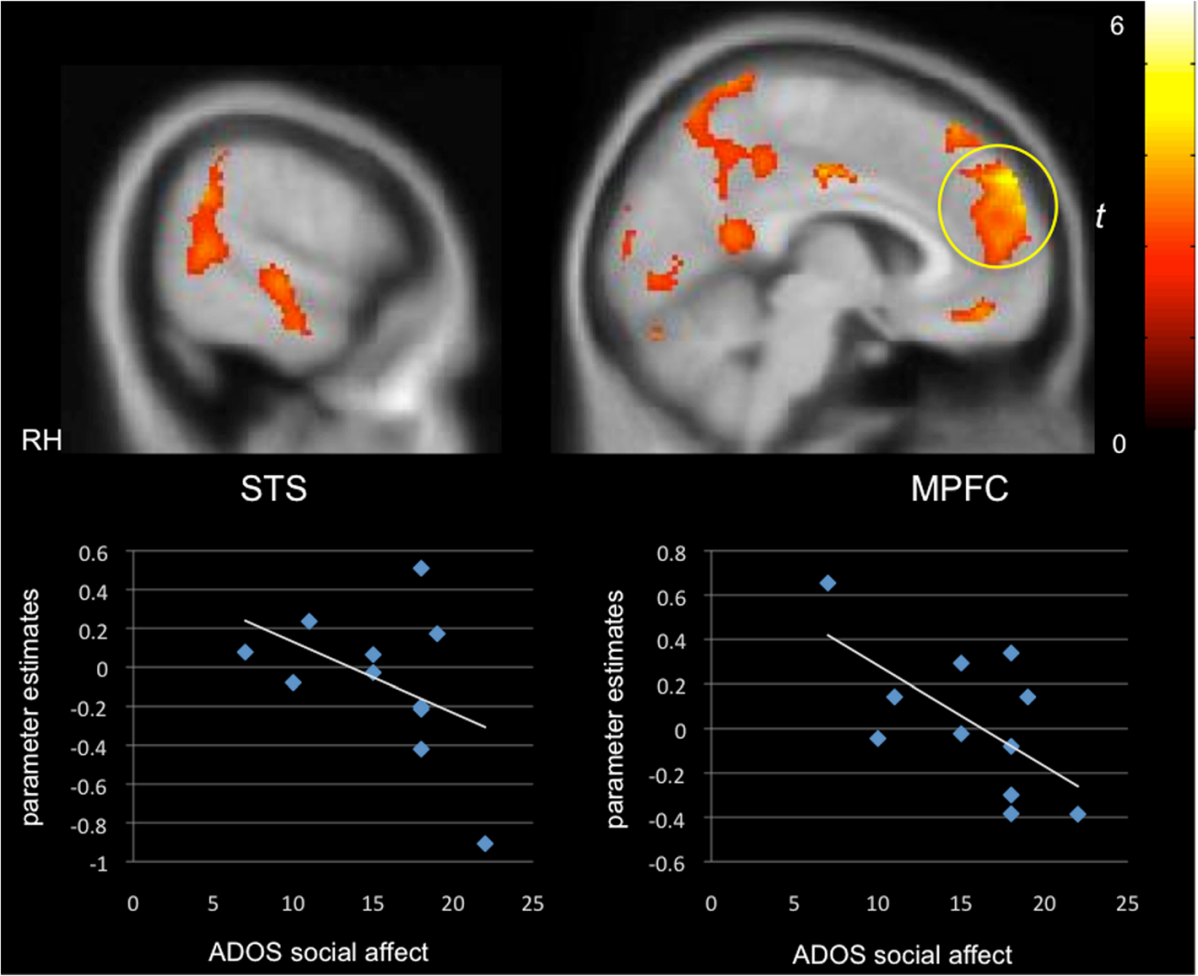
Selective activity for communicative vs. non-communicative sounds as a function of autism symptom severity in the social communication domain. A negative correlation was found in children with PMS between preferential activity in social brain networks and social symptom severity as measured by the social affect subscale of the ADOS-2 (*p* < 0.05, *k* > 194, corrected)

**Fig. 4:**
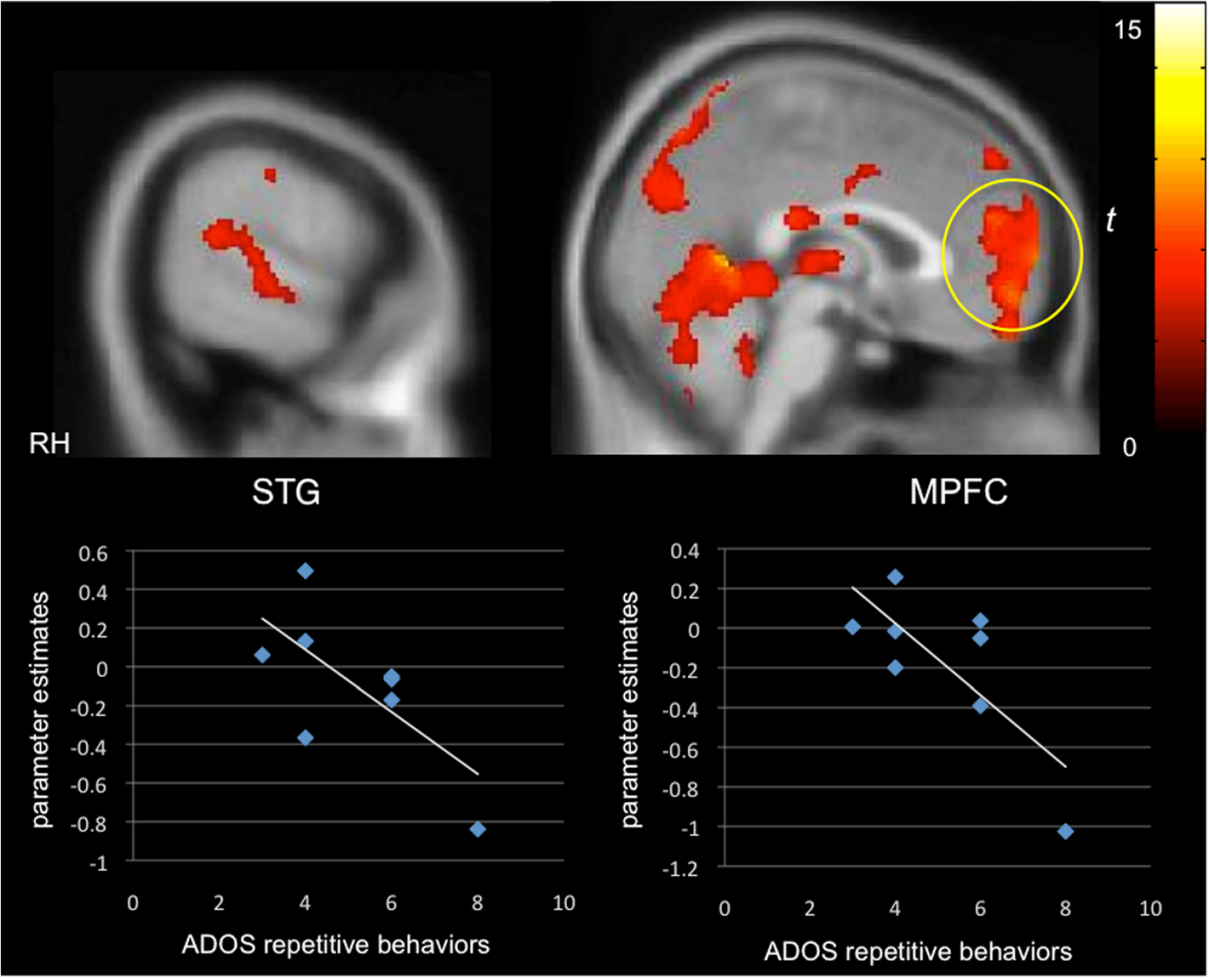
Selective activity for communicative vs. non-communicative sounds as a function of repetitive behaviors. A negative correlation was found in the iASD group between selective activity in the STG and MPFC and the repetitive behavior subscale of the ADOS (*p* < 0.05, *k* > 194, corrected)

We next probed the extent to which clinical characteristics were associated with neural selectivity for communicative sounds. Whole-brain regression analysis revealed that better orienting toward social sounds in the social orienting task was positively correlated with activity in the STG/STS (*x =* 54, *y =* −28, *z =* −4; *Z =* 2.59, *k* = 580) as well as in the MPFC (*x =* 0, *y =* 52, *z =* −4; *Z =* 3.60, *k =* 1587) and PCC (*x =* −4, *y =* −24, *z =* 40; *Z =* 2.89, *k =* 1813), relevant for theory of mind, in the PMS group (Fig. 2). Activity in these same regions was negatively correlated with autism symptom severity in the social affect domain of the ADOS (STG/STS: *x =* 58, *y =* −18, *z =* −4; *Z =* 2.50, *k =* 764; MPFC: *x =* −4, *y =* 40, *z =* 32; *Z =* 3.91, *k =* 4402; PCC: *x =* −16, *y =* −56, *z =* 30; *Z =* 2.83, *k =* 3363; Fig. 3), that is, greater activity in these “social brain” regions was associated with fewer autism symptoms in the social domain. While we expected to see a similar relationship between social symptoms and selective activity in the iASD group, instead, we found that activity in the right STG (*x =* 64, *y =* −42, *z =* 16; *Z =* 2.49, *k =* 658) and MPFC (*x =* −8, *y =* 56, *z =* 6; *Z =* 3.93, *k =* 4159) was inversely correlated with the repetitive behavior subscale of the ADOS (Fig. 4). For data visualization purposes, mean parameter estimates of activity were extracted during communicative vs. non-communicative sounds from the STG and MPFC clusters resulting from the whole-brain regression analyses within each group.

We also examined the relationship between receptive and expressive language levels, as measured by the MSEL, and preferential activity for communicative sounds. In children with PMS, selective MPFC activity (*x =* 18, *y =* 54, *z =* 6; *Z =* 2.81, *k =* 435) was positively correlated with receptive language scores, whereas in children with iASD, greater MPFC activity for communicative vs. non-communicative sounds (*x =* −14, *y =* 60, *z =* −6; *Z =* 3.64, *k =* 817) was associated with higher expressive language scores (Additional file 2: Figure S2).

## Discussion

This is the first controlled neuroimaging study in PMS. We found that despite severe impairments in language and social interaction, communicative vocalizations (infant- and adult-directed speech and communicative non-speech sounds, such as laughter) elicited greater activation in the right STG than non-communicative vocalizations (e.g., coughs and yawns) in the PMS group only. Selective activation in the STG for communicative sounds has previously been observed in typical adults and interpreted as neural infrastructure for recognition of the communicative significance of vocal sounds [19]. In contrast, here, the iASD group showed significant STG activity in response to non-communicative vocalizations but did not show differential STG activity in response to communicative sounds. This finding is consistent with a previous report that adults with ASD did not show voice-selective activity in the STG despite showing a normal response to non-vocal sounds [26].

These data suggest that the selective recruitment of STG for communicative sounds was relatively more intact in the PMS group than the iASD group. However, the PMS group showed selective STG activation for communicative vocalizations in the right hemisphere only, whereas previous studies with healthy adults have observed selective STG activity bilaterally [19, 26]. Given the severe deficits in language development and social communication that characterize PMS, sensitivity of the right STG to the communicative significance of vocalizations is noteworthy yet clearly insufficient for successful communication at the behavioral level. Lack of differential STG response in the left hemisphere may reflect some of the observed deficits in language and communication in children with PMS.

Previous studies using this and similar tasks have demonstrated that the STS/STG is the critical region for detecting the salience of communicative sounds [18, 19, 26]. In the PMS group, communicative vocalizations elicited greater activity than non-communicative vocalizations not only in the right STG but also in other “social brain” areas such as the MPFC and PCC, associated with theory of mind and critical nodes of the default mode network [35]. Furthermore, greater selective activity in the STG and MPFC was associated with fewer autism symptoms in the social communication domain and better orienting to social stimuli. Children with PMS showed lower rates of orienting to both social and non-social stimuli relative to those reported previously by Dawson et al. [31] for typically developing (TD) children and children with developmental delays (DD). This was not unexpected given that DQ and language levels were much lower in our PMS sample than for the groups sampled by Dawson and colleagues. However, interestingly, children with PMS were equally likely to orient to social and non-social stimuli. In contrast, Dawson et al. found that children with ASD were more likely to fail to orient to social than non-social stimuli and that, furthermore, this discrepancy was significantly greater for children with ASD than for TD and DD children. Here, the finding that the PMS group showed a ratio of social to non-social orienting comparable to that reported previously for children without autism [31] is consistent with neural selectivity for communicative vocalizations in spite of severe social communication deficits.

In the iASD group, individual differences in selective STG and MPFC activity were inversely related to autism symptomatology in the repetitive behavior domain. In addition, communicative vocalizations elicited selective activity in more posterior visual and subcortical regions. Previous studies have found that children with ASD orient less to social stimuli such as faces and voices and prefer a non-speech analog to motherese [24, 31, 36]. Lack of selective activity in the STG for communicative sounds may reflect a bias toward non-communicative sounds, which could be associated with repetitive behaviors and restricted interests. Moreover, recruitment of visual regions outside of typical language networks is consistent with previous fMRI studies with average IQ ASD samples that have observed atypical engagement of visual networks during language and social tasks [37, 38]. Although we initially expected that STG activity might be correlated with social communicative symptoms in both groups, we observed instead that selective “social brain” activity was linked to the repetitive behavior subscale in the iASD group. This finding could suggest that for children with iASD, who showed more repetitive behaviors than children with PMS on the ADOS and ADI, repetitive behaviors compete with social attunement and the development of neural selectivity for communicative and social stimuli; conversely, lack of neural selectivity for communicative signals could be associated with the development of repetitive behaviors in the absence of attention to and preference for social communicative stimuli. These interpretations are of course speculative as we did not follow participants over time.

In both groups, language skills (receptive language for PMS and expressive for iASD) were positively correlated with selective activity in the MPFC during communicative vs. non-communicative sounds. Previous studies have successfully used passive listening tasks with participants under sedation to map language-specific STG activity in infants, children with ASD, and those with language delay [39–41]. Accordingly, we expected that language level might be associated with preferential STG activity in one or both groups given that adult- and infant-directed speech are two of the communicative sound categories presented in the task. However, the speech presented consisted of Japanese words spoken by female native Japanese speakers to ensure that semantic comprehension would not be a confound. There is evidence to suggest that the MPFC plays a role in interpreting a speaker’s communicative intent beyond the literal meaning of the words used [42, 43] and that children with ASD show less activity in the MPFC than TD children when interpreting communicative intent is required [44]. Perhaps here, selective MPFC activity for communicative sounds is associated with better language skills in both groups because children with more developed language are more likely to detect the communicative intentions behind unfamiliar speech sounds at both the behavioral and neural levels.

This study had several limitations. First, while we were able to detect significant activity within each group as well as differences between groups, our small sample size leaves open the possibility that the findings may not generalize to the broader population of children with PMS and iASD. However, PMS is a rare genetic disorder, and this is a preliminary study comparing the neural functioning of children with PMS and iASD . Replication of these results will be important in future studies with larger samples.

Another limitation is that the two groups were imperfectly matched. For cognitive abilities, DQ was slightly higher in children with iASD (*p* = 0.13), and the groups were not matched on language age equivalents or sex ratio. The difference in DQ was not significant, and entering DQ as a covariate in the analyses did not substantially change the results. With respect to language levels, although both groups were minimally verbal, children with PMS had significantly lower receptive and expressive language age equivalents than children with iASD. We found that higher receptive and expressive language levels in the PMS and iASD groups, respectively, were associated with greater selective activity for communicative sounds in the MPFC, but not STG or other “social brain” regions. This suggests that lower language levels in the PMS group are unlikely to account for the between-group differences observed, since lower language levels should make it more likely for the PMS group to show less selective MPFC activity than the iASD group, rather than the opposite. With respect to gender, whereas the iASD group was all male, the PMS group was 45 % female, reflecting the higher male-to-female ratio in ASD and roughly equal sex ratio in PMS. A recent large study of voice-selective activity in typically developing men and women found highly similar temporal activation patterns in both groups for vocal vs. non-vocal sounds [21]. While little is known about gender effects on brain activity in ASD and PMS, the findings of Ahrens and colleagues [21] could suggest that gender differences are unlikely to explain the between-group differences in the right STG in the present study.

An additional limitation is related to sedation. While the severe intellectual disability in both groups necessitated the use of sedation in order for the participants to undergo scanning, ethical constraints prevented us from obtaining data on a typically developing control group. Although sedation is associated with reduced magnitude and spatial extent of temporal and frontal activity during auditory processing [45, 46], the passive listening task did elicit activity in primary auditory cortices in both groups in spite of propofol sedation. However, it is possible that sedation may affect the processing of PMS and iASD groups differently. We also cannot rule out the possibility that sedation results in a neural response that would not be detected in an awake state, yet previous findings of diminished frontal and temporal activity suggest that it is more likely that the activity detected would be even stronger without sedation [45, 46]. Finally, while significant differential activity in the right STG in the PMS group may suggest some degree of neural sensitivity to the communicative significance of some vocal sounds, conclusions cannot be made about whether the signal intensity and spatial extent of this activation are comparable or reduced relative to typically developing children.

## Conclusions

This pilot study is a first step toward characterizing the neural systems underlying social communication in PMS. Despite shared behavioral features, including comparable levels of autism symptom severity, children with PMS differed significantly from children with iASD in their neural response to communicative auditory signals. Whereas the PMS group showed selective activity for communicative relative to non-communicative vocalizations in the right STG and other social brain regions, the iASD group did not. Moreover, the relationship between clinical characteristics and neural selectivity was different between the two groups, suggesting that shared ASD features may partially reflect different functional neural abnormalities. This study is the first to examine brain function in PMS using a control group, but much more work with larger samples is needed to identify the neural underpinnings of PMS. Understanding both the neurodevelopmental consequences of *SHANK3* deficiency and the neural mechanisms associated with the social and cognitive deficits observed will help parse the heterogeneity in ASD. Moreover, the creation of an accurate brain phenotype will pave the road for developing new pharmacological strategies for targeting affected networks and may be important for identifying biomarkers of treatment response.

## Additional files

Additional file 1: Figure S1. Brain regions more strongly selective for communicative vocalizations in children with PMS relative to children with iASD. Greater neural selectivity was observed in the right STG, MPFC, left IFG, among other areas, in the PMS vs. iASD group (*p* < 0.05, *k* > 194, corrected). (PNG 212 kb) Additional file 2: Figure S2. Selective activity for communicative vs. non-communicative sounds as a function of language level. A positive correlation was observed between preferential MPFC activity and MSEL receptive language scores in the PMS group (left) and expressive language level in the iASD group (*p* < 0.05, *k* > 194, corrected). (PNG 260 kb)
