# Erratum to: Neural selectivity for communicative auditory signals in Phelan-McDermid syndrome

**DOI:** 10.1186/s11689-016-9143-z

**Published:** 2016-03-15

**Authors:** A. Ting Wang, Teresa Lim, Jesslyn Jamison, Lauren Bush, Latha V. Soorya, Teresa Tavassoli, Paige M. Siper, Joseph D. Buxbaum, Alexander Kolevzon

**Affiliations:** Seaver Autism Center for Research and Treatment, Icahn School of Medicine at Mount Sinai, One Gustave L. Levy Place, Box 1230, New York, NY 10029 USA; Department of Psychiatry, Icahn School of Medicine at Mount Sinai, New York, NY USA; Department of Neuroscience, Icahn School of Medicine at Mount Sinai, New York, NY USA; Friedman Brain Institute, Icahn School of Medicine at Mount Sinai, New York, NY USA; Department of Psychiatry, Rouge Valley Health System, Toronto, Canada; Department of Communication Sciences and Disorders, Northwestern University, Evanston, IL USA; Rush University Medical Center, Chicago, IL USA; Department of Genetics and Genomic Sciences, Icahn School of Medicine at Mount Sinai, New York, NY USA; Mindich Child Health and Development Institute, Icahn School of Medicine at Mount Sinai, New York, NY USA; Department of Pediatrics, Icahn School of Medicine at Mount Sinai, New York, NY USA

After publication of this study [1], we noticed that Table 1 contained formatting inconsistencies and a missing value due to a copyediting error. The publisher apologizes for any inconvenience caused. Please see the corrected Table 1 below:

**Table 1:** Participant characteristics

	PMS mean (SD)	iASD mean (SD)	*p* value
Age, years	7.9 (3.9)	9.5 (3.9)	0.37
DQ	21 (14)	35 (25)	0.13
Language quotient	17 (14)	31 (23)	0.13
Nonverbal quotient	24 (15)	39 (28)	0.15
Mullen receptive language AE (months)	14 (10)	30 (9)	0.001
Mullen expressive language AE (months)	11 (9)	25 (12)	0.005
ADOS total	18 (6)	20 (4)	0.43
ADOS social affect	16 (5)	15 (3)	0.72
ADOS repetitive behavior	3 (2)	5 (2)	0.007
ADI communication	12 (3)	13 (5)	0.27
ADI social interaction	20 (7)	24 (5)	0.16
ADI repetitive behavior	4 (2)	7 (2)	0.02
Vineland adaptive behavior composite	52 (11)	55 (10)	0.59

*SD* standard deviation, *DQ* developmental quotient, *Mullen* Mullen Scales of Early Learning, *AE* age equivalent, *ADOS* Autism Diagnostic Observation Schedule, Second Edition, *ADI* Autism Diagnostic Interview, Revised

## References

[1] Wang AT, Lim T, Jamison J, Bush L, Soorya LV, Tavassoli T, Siper PM, Buxbaum JD and Kolevzon A. Neural selectivity for communicative auditory signals in Phelan-McDermid syndrome. J Neurodev Disord. 2016;8:5. 10.1186/s11689-016-9138-9 PMC4763436 26909118

