# Supplementary figures and images for: Neural selectivity for communicative auditory signals in Phelan-McDermid syndrome
Source: J Neurodev Disord. 2016 Feb 23;8:5. doi: 10.1186/s11689-016-9138-9 (PMC4763436; doi:10.1186/s11689-016-9138-9)

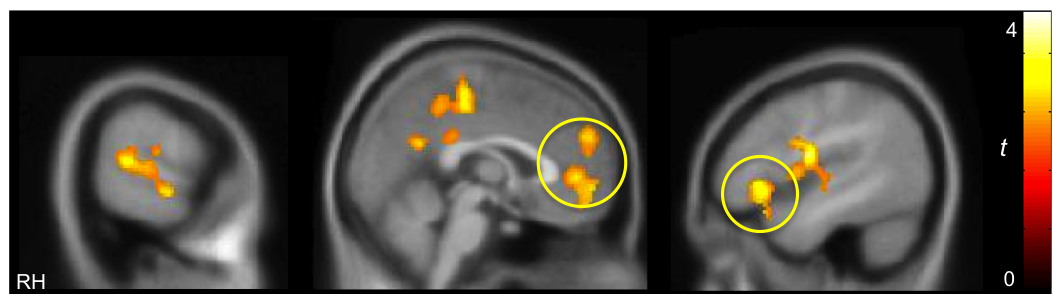

Supplement: Additional file 1: Figure S1. — Brain regions more strongly selective for communicative vocalizations in children with PMS relative to children with iASD. Greater neural selectivity was observed in the right STG, MPFC, left IFG, among other areas, in the PMS vs. iASD group (p < 0.05, k > 194, corrected). (PNG 212 kb) [file 11689_2016_9138_MOESM1_ESM.png]

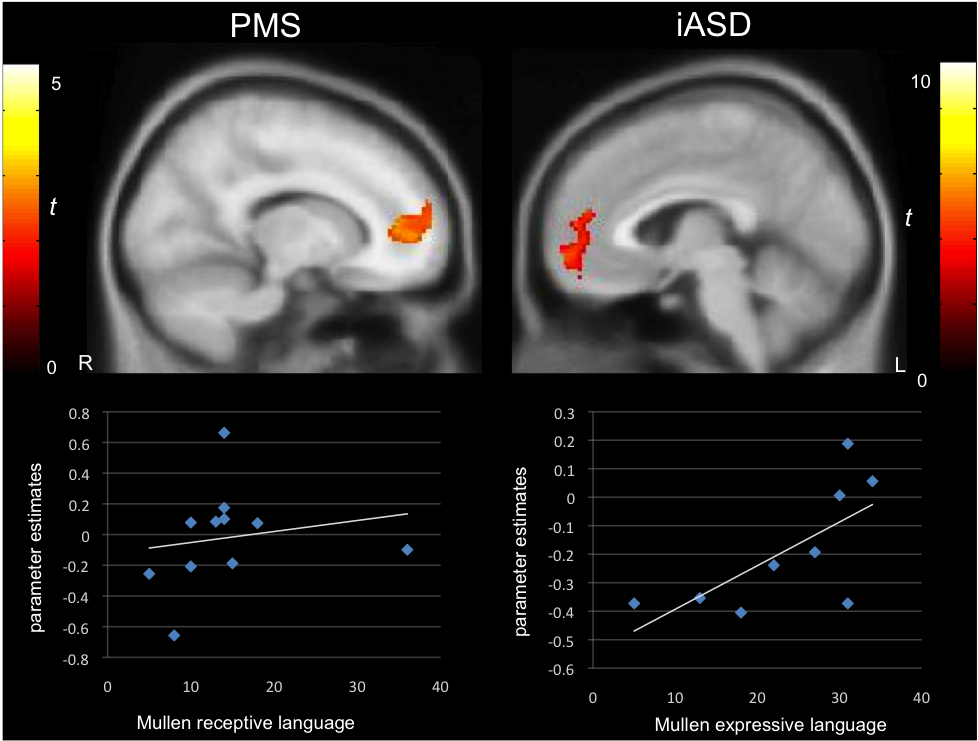

Supplement: Additional file 2: Figure S2. — Selective activity for communicative vs. non-communicative sounds as a function of language level. A positive correlation was observed between preferential MPFC activity and MSEL receptive language scores in the PMS group (left) and expressive language level in the iASD group (p < 0.05, k > 194, corrected). (PNG 260 kb) [file 11689_2016_9138_MOESM2_ESM.png]
